# Effects of COVID-19 Pandemic on the Lived Experience of Diverse Older Adults Living Alone With Cognitive Impairment

**DOI:** 10.1093/geroni/igaa057.3454

**Published:** 2020-12-16

**Authors:** Elena Portacolone, Anna Chodos, Jodi Halpern, Kenneth Covinsky, Sahru Keiser, Jennifer Fung, Elizabeth Rivera, Julene K Johnson

**Affiliations:** 1 University of California San Francisco, San Francisco, California, United States; 2 University of California Berkeley, Berkeley, California, United States; 3 UCSF, San Francisco, California, United States

## Abstract

Background and Objectives: Even before the COVID-19 pandemic, older adults with cognitive impairment living alone (an estimated 4,3 million individuals in the United States) were at high risk for negative health outcomes. There is an urgent need to learn how this population is managing during the pandemic. Research Design and Methods: This is a qualitative study of 24 adults aged 55 and over living alone with cognitive impairment from diverse racial/ethnic backgrounds. Participants’ lived experiences during the pandemic were elicited via 59 ethnographic interviews conducted over the phone either in English, Spanish, or Cantonese. Using a qualitative content analysis approach, interview transcripts and fieldnotes were analyzed to identify codes and themes. Results: Qualitative analysis of transcripts revealed five themes: 1) fear generated by the pandemic; 2) distress stemming from feeling extremely isolated; 3) belief in misinformation, 4) strategies for coping during the pandemic; and 5) the importance of access to essential services. Discussion and Implications: This pandemic put a spotlight on the precarity and unmet needs of older adults living alone with cognitive impairment living. Findings underscore the need to expand access to home care aides and mental health services for this population.

